# Regulatory Landscapes of Non-Coding RNAs During Drought Stress in Plants

**DOI:** 10.3390/ijms26209892

**Published:** 2025-10-11

**Authors:** Paulina Bolc, Marta Puchta-Jasińska, Adrian Motor, Marcin Maździarz, Maja Boczkowska

**Affiliations:** 1 Plant Breeding and Acclimatization Institute-National Research Institute, 05-870 Błonie, Poland; a.motor@ihar.edu.pl (A.M.); m.boczkowska@ihar.edu.pl (M.B.); 2 Institute of Fundamental Technological Research Polish Academy of Sciences, 02-106 Warsaw, Poland; mmazdz@ippt.pan.pl

**Keywords:** non-coding RNA, drought, cereals, miRNA, siRNA, circRNA, lncRNA

## Abstract

Drought is a leading constraint on plant productivity and will intensify with climate change. Plant acclimation emerges from a multilayered regulatory system that integrates signaling, transcriptional reprogramming, RNA-based control, and chromatin dynamics. Within this hierarchy, non-coding RNAs (ncRNAs) provide a unifying regulatory layer; microRNAs (miRNAs) modulate abscisic acid and auxin circuits, oxidative stress defenses, and root architecture. This balances growth with survival under water-deficient conditions. Small interfering RNAs (siRNAs) include 24-nucleotide heterochromatic populations that operate through RNA-directed DNA methylation, which positions ncRNA control at the transcription–chromatin interface. Long non-coding RNAs (lncRNAs) act in cis and trans, interact with small RNA pathways, and can serve as chromatin-associated scaffolds. Circular RNAs (circRNAs) are increasingly being detected as responsive to drought. Functional studies in *Arabidopsis* and maize (e.g., ath-circ032768 and circMED16) underscore their regulatory potential. This review consolidates ncRNA biogenesis and function, catalogs drought-responsive modules across model and crop species, especially cereals, and outlines methodological priorities, such as long-read support for isoforms and back-splice junctions, stringent validation, and integrative multiomics. The evidence suggests that ncRNAs are tractable entry points for enhancing drought resilience while managing growth–stress trade-offs.

## 1. Introduction

Drought is one of the most pervasive constraints on plant growth and yield, and its global impact is expected to worsen with continued warming. Recent analyses indicate that warming accelerates the severity of meteorological and agricultural droughts worldwide. Moreover, sector-specific assessments emphasize the increasing risks to crop production and food security [1,2].

**Figure 1:**
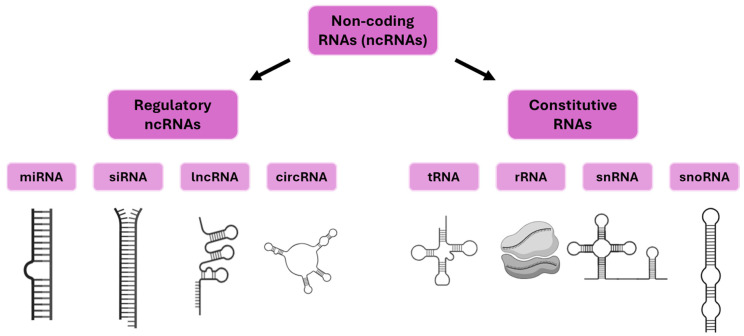
Classification of non-coding RNAs in plants.

The molecular mechanisms underlying plant responses to drought stress have been shown to operate through an integrated network of signaling, gene regulation, and metabolic adaptations involving hormones, transcription factors, redox, and osmotic pathways. A distinctive feature of the drought response is the rapid accumulation of the abscisic acid (ABA) phytohormone, which plays a pivotal role in regulating stomatal closure, minimizing water loss, reprogramming transcription through the binding of transcription factors to ABA-responsive elements (AREB/ABF) and marker genes such as *RD29A* and *RD22*, and *encoding 9-cis-epoxycarotenoid dioxygenase* (*NCED3*). In addition to the aforementioned, other hormones, including jasmonic acid (JA), salicylic acid (SA), ethylene, and auxin, have been shown to coordinate or antagonize the expression of drought-adaptive genes, thereby further enhancing or refining responses [3,4]. The main classes of drought marker genes are known to be activated or inhibited by major transcription factors such as DREB (dehydration-responsive element binding), NAC, MYB, HD-ZIP, bZIP, and WRKY, which have been shown to integrate ABA-dependent and ABA-independent signaling pathways [3,5]. These transcriptional cascades regulate genes encoding late embryogenic abundant (LEA) proteins, dehydrins, aquaporins, enzymes involved in the biosynthesis of osmo-protective and compatible solutes (e.g., proline, trehalose, raffinose family oligosaccharides), detoxification proteins (Reactive Oxygen Species scavengers such as superoxide dismutase, ascorbate peroxidase, and catalase), and various channel proteins crucial for water balance [3,4]. Drought also has a significant impact on photosynthetic efficiency. This is due to a reduction in Rubisco activity, impairment to photosystem II function, and alteration to Calvin cycle fluxes [4]. All of these factors are linked to further regulatory changes in stress marker genes and metabolic enzymes. At the biochemical level, increased levels of certain amino acids (particularly proline), polyamines, sugars, and secondary metabolites contribute to stress tolerance through the maintenance of osmotic balance, membrane stability, and ROS detoxification [3,4]. In parallel, mitogen-activated protein kinase (MAPK) and calcium-dependent protein kinase (CDPK) cascades, as well as SnRK2 kinases, function as signal integrators, linking stress sensing to the activation of gene regulatory circuits, metabolic adaptation, and communication with other signaling networks [3]. Epigenetic modifications, including dynamic changes in histone methylation (e.g., H3K4me3 in drought loci), DNA methylation changes, and transcriptional memory, further fine-tune gene expression and maintain adaptive potential in various stress situations Recent advances have revealed that gene expression during the drought response is regulated at multiple levels, including transcriptional, posttranscriptional, and chromatin remodeling processes. This ensures flexibility and specificity in stress adaptation [6,7]. The complexity is further compounded by the involvement of non-coding RNAs (ncRNAs). Non-coding RNAs are classified into two primary categories: regulatory ncRNAs and constitutive (housekeeping) ncRNAs (Figure 1) [8]. Constitutive ncRNAs include transfer RNAs (tRNAs), ribosomal RNAs (rRNAs), small nuclear RNAs (snRNAs), and small nucleolar RNAs (snoRNAs). These ncRNAs are stably expressed and essential for fundamental processes such as translation, RNA splicing, and ribosome biogenesis [9]. Regulatory non-coding RNAs encompass microRNAs (miRNAs), small interfering RNAs (siRNAs), long non-coding RNAs (lncRNAs), and circular RNAs (circRNAs) [10]. These regulatory ncRNAs modulate drought stress responses by targeting genes and regulatory networks associated with stress, including those involved in hormone biosynthesis and signaling pathways, transcription factors, and metabolic processes essential for water conservation and stress tolerance [6,11]. Genome-wide transcriptomic analyses of various plant species, including *Arabidopsis*, maize, rice, and wheat, have identified numerous drought-responsive ncRNAs, highlighting their conserved and species-specific functions [6].

This review aims to provide a comprehensive synthesis of recent discoveries regarding ncRNA-mediated regulation in plant responses to drought stress. Specifically, this review harmonizes terminology and biogenesis frameworks across the microRNA, small interfering RNA, long non-coding RNA, and circular RNA classes. This study provides evidence for the non-coding RNA-guided regulation of hormone signaling, development, redox, and chromatin states during drought. This review also highlights functional case studies with genetic validation across model and crop species. This review outlines the methodological standards and knowledge gaps that limit the translation of research into the breeding and engineering of climate-resilient cultivars. By consolidating these topics, this review aims to provide a consistent reference for designing experiments and prioritizing ncRNA targets for drought adaptation.

## 2. Classification and Biogenesis of Non-Coding RNAs (ncRNAs)

Non-coding RNAs are a diverse group of RNA molecules that do not encode proteins. In plants, ncRNAs can be classified on the basis of their length, structure, and location in the genome. There are three main categories of ncRNAs: short ncRNAs (sRNAs), long ncRNAs, and circular RNAs. Furthermore, the process of their biogenesis, which involves various RNA polymerases, processing enzymes, and auxiliary factors that confer specificity to each class, is of equal importance. The integration of classification with biogenetic pathways furnishes a coherent framework for comprehending the manner in which non-coding RNAs contribute to the regulation of plant development and adaptation to stress.

### 2.1. Small Non-Coding RNAs (sRNAs)

sRNAs are 20–24 nucleotide (nt) regulators that guide Argonaute (AGO) proteins to RNA or chromatin. These sRNAs shape posttranscriptional gene silencing (PTGS) and transcriptional gene silencing (TGS/RdDM) [12]. The two major classes are microRNAs and small interfering RNAs.

#### 2.1.1. MicroRNAs (miRNAs)

Plant miRNAs are a class of small, endogenous, non-coding RNAs that are typically 20–22 nucleotides in length. They mediate posttranscriptional gene silencing and play an important role in development, stress responses, and nutrient homeostasis [12,13,14]. Plant miRNAs are formed when MIR genes are transcribed by RNA polymerase II (Pol II) resulting in primary miRNA transcripts (pri-miRNAs) with a distinctive stem-loop secondary structure, often capped and polyadenylated [15,16]. In the cell nucleus, these pri-miRNAs are processed in specialized subnuclear compartments called dicing bodies (D-bodies). There, a microprocessor complex composed of Dicer-like 1 (DCL1), Hyponastic Leaves 1 (HYL1), and Serrate (SE) cuts the loops of precursor miRNAs (pre-miRNAs) and generates a miRNA/miRNA* duplex. The duplex is stabilized by 2′-O-methylation via HEN1 at the 3′ ends, protecting it against uridylylation and degradation [15,16,17].The evidence supports the nuclear loading model, in which mature miRNAs are loaded into AGO1 in the nucleus and AGO1-miRNA complexes are exported to the cytoplasm via CRM1/Exportin-1. Perturbing this route (e.g., by pathogen effectors) compromises silencing [15,16,18,19]. HASTY (the EXPORTIN-5 ortholog) modulates miRNA levels and long-distance movement and links *MIR* transcription with processing. However, it is not a cytoplasmic “maturation” step [20,21]. Guide-strand selection from the miRNA/miRNA* duplex follows general principles: the strand with the less stable 5′ end and the appropriate features is preferentially retained as the guide strand, whereas the passenger strand is typically discarded. AGO preferences also play a role (e.g., AGO1 favors a 5′ uridine) [15]. Many miRNA* species are degraded, but some accumulate and can be loaded into AGOs in specific contexts [15,16]. Once formed, plant miRISCs act predominantly by endonucleolytic cleavage of target mRNAs with extensive complementarity and/or translational repression. The relative contribution depends on the target context and developmental or stress conditions [10]. These findings outline a nucleus-centered, condensate-enabled pathway that couples *MIR* transcription, pri-miRNA processing, HEN1-mediated terminal protection, nuclear AGO1 loading, and CRM1-dependent export to the cytoplasm. Therefore, miRISCs execute posttranscriptional gene silencing. Some 22-nt miRNAs initiate the biogenesis of RDR6-dependent secondary small interfering RNAs known as phased siRNAs (phasiRNAs) [22]. The systemic mobility of select microRNAs (miRNAs), as demonstrated by the shoot-to-root translocation of miR399 in phosphate signaling, further expands the functional scope of miRNA regulatory networks [23]. Genetic, transcriptomic, and molecular studies have revealed a nucleus-centered, highly regulated pathway that unites MIR transcription, stem-loop processing, HEN1 stabilization, AGO1 loading, nuclear export, and cytoplasmic activity. This pathway ensures precise and robust posttranscriptional gene regulation in plants [24].

#### 2.1.2. Small Interfering RNA (siRNA)

Plant siRNAs are a diverse group of double-stranded RNAs consisting of 20–24 nucleotides. They play crucial roles in posttranscriptional gene silencing, genome defense, and epigenetic regulation in plants [8,22]. Traditionally, siRNAs are classified into three principal subclasses based on their origin and mode of action: heterochromatic siRNAs (hc-siRNAs), trans-acting siRNAs (ta-siRNAs), and phased siRNAs. The biogenesis of plant small interfering RNAs relies on the activities of RNA-dependent RNA polymerase (RDR) and Dicer-like (DCL) enzymes. Typically, RNA polymerase IV (Pol IV) and/or Pol II transcribe precursor RNAs from transposons, repetitive elements, or specific coding or non-coding genes. For many siRNA forms, including hc-siRNAs and epigenetically activated small interfering RNAs (easiRNAs), Pol IV-derived transcripts are converted into double-stranded RNAs (dsRNAs) by RDR2 in the nucleus. Then, DCL3 processes these dsRNAs into 24-nt siRNAs, which load into AGO4/6/9 [22,25,26]. These siRNAs guide sequence-specific DNA methylation and transcriptional silencing at cognate loci through the RNA-directed DNA methylation (RdDM) pathway. Notably, hc-siRNAs are essential for maintaining genome stability by silencing transposable elements and repetitive sequences [22,26,27]. Ta-siRNAs, notably from TAS gene loci, arise from Pol II transcripts that are initially cleaved by AGO1-loaded miRNAs (often 22-nt triggers). The resulting cleavage fragment is converted into dsRNA via RDR6 and SGS3. This dsRNA is then processed by DCL4 into 21-nt siRNAs in a precisely phased manner. These ta-siRNAs act in trans to direct the sequence-specific cleavage of nonhomologous target mRNAs, which is an amplification mechanism for gene silencing [22,28]. Specific coding or non-coding loci known as PHAS genes or regions are the sources of phasiRNAs. They are generated by miRNA-guided slicing, typically by 22-nt miRNAs, and proceed with RDR6-dependent dsRNA synthesis and DCL4/2-catalyzed production of siRNAs at regular intervals along the precursor. PhasiRNAs and tasiRNAs fundamentally expand the regulatory impact of miRNAs by generating secondary siRNA populations that can target various loci [22]. Unlike miRNAs, plant siRNAs typically require perfect or near-perfect complementarity to recognize their targets. They guide DNA methylation or mRNA cleavage through AGO1/AGO4/AGO6 complexes, depending on the siRNA subclass. Furthermore, siRNAs exhibit cell-to-cell and long-distance mobility. Plasmodesmata and phloem pathways facilitate their systemic signaling roles [12,29]. At the *Arabidopsis SRO5–P5CDH* locus, salt stress induces a DCL2-dependent primary nat-siRNA that engages RDR6/SGS3 to generate DCL1-dependent secondary 21-nt nat-siRNAs. Pol IV/NRPD1A contributes to this specific system [22,30]. The resulting siRNA duplexes are 2′-O-methylated at their 3′ ends by HEN1 across pathways, which protects them from tailing and trimming [31]. The duplexes are loaded into Argonaute proteins according to size and 5′ nucleotide preferences (e.g., AGO4/6 selects 24-nt, 5′ A-enriched siRNAs). This is followed by eliminating the passenger strand [22,32,33]. Many 21/22-nt siRNA-AGO complexes act in the cytoplasm to direct mRNA cleavage. However, 24-nt AGO4-clade complexes engage nascent scaffold RNAs at target loci in the nucleus to reinforce RNA-directed DNA methylation. Assembly can involve cytoplasmic loading with subsequent nuclear import [22,32,33].

### 2.2. Long Non-Coding RNA (lncRNA)

LncRNAs are defined as regulatory transcripts longer than 200 nt that lack protein-coding capacity and exist in a diverse array of forms, many of which resemble messenger RNAs in their processing and structure, i.e., are capped, spliced, and polyadenylated, however some lack poly(A) tails and/or a 7-methyl-G cap. They regulate gene expression and genome stability across development and environmental responses [34,35]. Most plant lncRNAs, including those in the intergenic, intronic, and antisense classes, are transcribed by Pol II are processed through canonical pathways involving 5′ capping, splicing, and 3′ polyadenylation [10,34]. However, a subset is generated by plant-specific RNA polymerase V (Pol V) which transcribes chromatin-associated, nonpolyadenylated scaffold RNAs that recruit siRNA-AGO complexes to target loci, guiding RdDM and associated chromatin changes [27,36]. Recent cryo-EM structural studies have refined the understanding of Pol V-derived transcripts as a distinct, lncRNA-like class specialized for chromatin-associated functions [37]. The selection of polymerase and the specificity of the transcribed locus are determined by chromatin features and the action of dedicated recruitment factors. This emerging view delineates the unique structural and functional adaptations that enable Pol V to occupy and act upon targeted chromatin sites [36]. Nuclear and cytoplasmic pools reflect diverse sites of action [34,38]. From a structural perspective, plant lncRNAs can be categorized into various classes, including intergenic (lincRNAs), intronic, sense and antisense, divergent, and convergent. These classifications are determined by their genomic location, transcriptional orientation, and the relationship they have with neighboring genes [39]. The functional diversity of these non-coding RNA molecules is similarly broad in scope, encompassing both cis-acting (modifying transcription near the site of transcription) and trans-acting (modifying transcription at distant loci) processes, in addition to the modulation of transcription, chromatin state, and posttranscriptional processes. The key mechanisms include acting as molecular decoys (target mimics), sponges for miRNAs (competing endogenous RNA, ceRNA activity), scaffolds for recruiting chromatin modifiers, and even as precursors for smaller regulatory RNAs such as siRNAs and miRNAs [10,40]. For instance, the occurrence of canonical target mimicry in nutrient signaling is demonstrated by the sequestering of miR399 by the IPS1 lncRNA in *Arabidopsis*, thus modulating phosphate homeostasis [41]. In comparison to sRNA, the conservation of the sequence of lncRNAs is low, but the specificity for tissue, developmental stage, and environmental condition is high. The secondary structure and genomic context of lncRNAs contribute to conserved regulatory functions [10]. Cross-talk between lncRNAs and epigenetic pathways (e.g., chromatin remodeling, DNA methylation, histone modification) is increasingly recognized as central to coordinating plant development and stress adaptation. Therefore, plant lncRNAs function as a dynamic regulatory layer that interfaces with both the genetic and epigenetic architecture, thereby orchestrating gene expression dynamics from the nucleus to the cytoplasm [39].

#### 2.2.1. Long Intergenic ncRNAs (lincRNAs)

LincRNAs, which arise from intergenic loci, represent a significant subset of plant lncRNAs. RNA polymerase II transcribes most lincRNAs, carries 5′ caps and poly(A) tails, and results in low, tissue-specific expression and limited primary sequence conservation. They also frequently incorporate transposable-element-derived sequences [34,35,42]. LincRNAs can regulate nearby protein-coding genes in cis by influencing chromatin states and three-dimensional (3D) genome topology. They can also act in trans on distant targets [34,35,42]. Genome-scale surveys have revealed thousands of lincRNAs that respond to development and stress, and these lincRNAs exhibit extensive population-level variability driven by epigenetic silencing [42]. Functionally, APOLO coordinates auxin-responsive networks via R loops and chromatin modulation [43]. R loops are three-stranded nucleic acid structures that form when a newly synthesized RNA molecule binds to its complementary DNA template strand. This process displaces the non-template DNA strand and can affect the state of chromatin and local gene expression [44]. ARES shapes lateral root architecture [45]. Potato lincRNAs modulate proximal gene expression during plant–pathogen interactions [46].

#### 2.2.2. Intronic lncRNAs (incRNAs)

IncRNAs originate from the introns of protein-coding genes or stabilized intron-derived lariats [34,47]. In plants, most incRNAs are transcribed by RNA polymerase II, bear 5′ caps and poly(A) tails, and are typically low-abundance and condition specific. By recruiting chromatin regulators or modulating elongation and splicing, lncRNAs often regulate their host loci in cis. Some incRNAs operate in trans [34,35]. The *Arabidopsis* incRNA COLDAIR, which is generated from the long first intron of FLC, associates with polycomb repressive complex 2 (PRC2) to promote H3K27me3 and enforce vernalization-induced gene silencing [48]. In rice, the incRNA RIFLA, produced from the first intron of OsMADS56, represses its host gene and promotes flowering [49]. Stable intron lariats further expand the repertoire of intron-origin non-coding transcripts.

#### 2.2.3. Antisense lncRNAs (NATs)

NATs are endogenous RNA molecules transcribed from the DNA strand opposite a sense transcript (protein- or non-protein-coding), yielding sequences complementary to their sense RNAs. NATs can originate from the same genomic locus as their sense partner (cis-NATs) or from different loci (trans-NATs). They participate in diverse regulatory mechanisms, including RNA interference, alternative splicing, and epigenetic regulation. Thus, they influence gene expression across a variety of tissues and environmental contexts [50]. Antisense transcription can repress sense transcription through transcriptional interference and polymerase collision and interact with chromatin-based silencing pathways [51,52]. NATs may also affect splicing and create double-stranded RNA substrates that yield NAT-siRNAs under stress conditions. At the *Arabidopsis* FLC locus, COOLAIR provides a rapid, antisense-driven repression pathway operating in parallel with PRC2 dependent, epigenetic silencing during vernalization [51]. The cold-responsive SVALKA/asCBF1 module restrains CBF1 through antisense transcription and small RNA production [53,54]. In maize, the cis-NAT PILNCR2 interacts with PHT1 transcripts to form RNA:RNA duplexes, which antagonize the action of miR399 and promote phosphate acquisition [55]. Plant NATs constitute a versatile regulatory layer integrated with transcriptional, chromatin, and small RNA pathways [50,51,52].

#### 2.2.4. Sense lncRNAs

Sense long non-coding RNAs are transcripts from the same strand as neighboring protein-coding genes. They may overlap exons (sense-exonic) or originate within introns of host genes [56]. Sense lncRNAs influence the expression of their cognate loci through chromatin-linked processes, local three-dimensional (3D) architecture, transcriptional dynamics, and RNA processing, primarily by acting in cis [34,57]. In *Arabidopsis FLOWERING LOCUS C* (*FLC*), the sense incRNA COLDAIR and the promoter-proximal sense lncRNA COLDWRAP cooperate with Polycomb to establish a repressive chromatin state during vernalization [48,58]. This finding illustrates how sense lncRNAs can integrate environmental cues with gene regulation.

#### 2.2.5. Bidirectional lncRNAs

Bidirectional lncRNAs in plants originate from divergent transcription at shared promoters that generate opposite-facing transcription start site (TSS) pairs within a few hundred base pairs [59]. In *Arabidopsis*, mapping nascent transcripts and perturbation of RNA decay reveal widespread bidirectional promoters and validate the existence of bidirectional non-coding promoters during germination. Many of these RNAs are exosome sensitive, which explains their underrepresentation in steady-state RNA-seq [59,60,61]. Bidirectional initiation is associated with accessible chromatin and RNAPII occupancy; expression from paired TSSs may be coordinated or decoupled. Notably, unstable enhancer-derived RNAs are rarer in plants than in animals. However, promoter-proximal divergent initiation is prevalent, albeit context dependent [62]. Therefore, bidirectional lncRNAs extend promoter output and contribute to gene regulatory plasticity during development and stress.

### 2.3. Circular RNA (circRNA)

Plant circular RNAs (circRNAs) are covalently closed RNAs that typically exhibit enhanced stability, limited primary sequence conservation, and condition-specific expression [63]. Recent functional studies have implicated plant circRNAs in stress adaptation. For example, drought-responsive circRNAs are prevalent in maize roots, and overexpressing circMED16 increased drought tolerance in *Arabidopsis* [64]. In *Arabidopsis*, ath-circ032768 acts as a competing endogenous RNA that sequesters miRNA472 to increase RPS5, promoting drought resistance [65]. In addition to RNA–RNA crosstalk, emerging reports indicate that subsets of plant circRNAs may associate with ribosomes and undergo translation, although the extent and physiological relevance of these interactions remain to be established. Community resources such as PlantCircRNA curate circRNA annotations across dozens of plant species, enabling comparative analyses and target prioritization. Plant circRNAs form a flexible regulatory layer linked to gene regulation and environmental responses.

#### 2.3.1. Exonic circRNAs (ecircRNAs)

Exonic circular RNAs (ecircRNAs) are covalently closed RNAs that comprise only exonic sequences from a single locus [66]. Genome-scale profiling revealed that EcircRNAs constitute the majority of plant circRNAs and exhibit tissue- and condition-specific accumulation [67]. Functionally, EcircRNAs contribute to plant defense. For example, several exonic circles modulate responses to *Pseudomonas syringae* and Botrytis cinerea in *Arabidopsis*, acting synergistically with their linear counterparts [68]. One well-validated example is circGORK, which is produced from GORK exons. Its overexpression alters abscisic acid sensitivity and drought behavior, which is consistent with its role in stomatal regulation [66]. Community resources such as PlantCircRNA centralize circRNA annotations across dozens of species, enabling cross-species comparisons and hypothesis generation [69]. Plant circRNAs are covalently closed RNAs predominantly generated through back-splicing. In this process, a downstream 5′ splice donor joins an upstream 3′ splice acceptor to produce a back-splice junction. This circular topology lacks exposed 5′/3′ ends, which confers enhanced exonuclease resistance. Nevertheless, circRNAs typically accumulate at low levels in a tissue- and condition-specific manner [66,67]. Two nonexclusive models describe plant circRNA formation: The first is direct backsplicing, which involves intronic complementary sequences—often inverted repeats or transposable element-derived sequences—that base pair to juxtapose splice sites, favoring circularization over canonical linear splicing. This process produces ecircRNAs when intervening introns are removed or eiciRNAs when intronic segments are retained. The efficiency of direct back-splicing is modulated by cis-elements and trans-acting RNA-binding/splicing factors that adjust the balance between linear splicing and circularization [66,70]. Importantly, primate-specific Alu elements frequently drive intron pairing in mammals. However, Alu sequences are absent from plant genomes. In plants, other repeated or inverted intronic sequences play analogous roles in pairing [66,67]. The second model is exon-skipping/lariat-driven circularization. Alternative splicing can generate a lariat intermediate containing one or more exons. Subsequent processing of this lariat produces ecircRNAs or eiciRNAs, depending on intron retention. Genetic and transcriptomic evidence in *Arabidopsis* and crops indicates that stabilized lariat RNAs contribute to the plant circRNA repertoire. This balance is shaped by splicing dynamics, intron architecture, and RNA surveillance [66,69]. Recent long-read and curated atlas resources have improved discrimination among ecircRNAs, eiciRNAs, and intronic circRNAs across studies. These resources have also reduced false positives from short-read mapping and documented widespread, yet context dependent, circRNA expression across more than 90 plant species [67].

#### 2.3.2. Intronic circRNAs

Intronic circular RNAs (ciRNAs) are covalently closed RNAs composed entirely of intronic sequences from host loci [66,70]. Although ciRNAs appear to be less prevalent than exonic circles and show tissue- and condition-specific accumulation, systematic surveys and the PlantCircRNA knowledge base document ciRNAs across dozens of plant species [66,67,69]. Research suggests that plant ciRNAs are often nuclear and can regulate the expression of their parental genes in cis; however, direct functional demonstrations remain scarce. One notable example is an *Arabidopsis* lariat-derived ciRNA whose overexpression caused pleiotropic developmental phenotypes and global transcriptome changes, highlighting its regulatory potential at the host locus [71]. A stable, nuclear-skewed subset of plant circular RNAs with emerging roles in locus-proximal regulation and stress-associated expression programs is represented by ciRNAs.

#### 2.3.3. Exon–Intron circRNAs (eiciRNAs)

Exon–intron circular RNAs (eiciRNAs) are circular RNAs that contain intronic segments between circularized exons. EiciRNAs have been documented in plants across species, but they generally occur at a lower frequency than do exonic circles. They also display tissue- and stress-dependent accumulation with a nuclear bias [66,69,70]. Current syntheses focusing on plants implicate EiciRNAs mainly in cis-modulating their parental loci and chromatin-linked regulation. However, direct in-plant functional tests remain limited [66,70]. Community resources now catalog EiciRNAs alongside other circRNA classes, enhancing cross-species comparability and facilitating the identification of candidates for targeted experiments [69].

#### 2.3.4. Intergenic circRNAs

Intergenic circRNAs are circular transcripts whose back-splice junctions are located in intergenic regions, i.e., outside annotated genes [67,70]. They are consistently reported across plant species but usually constitute a minority relative to exonic circles and display tissue- and condition-specific accumulation [66,67,70]. Their circular topology confers enhanced stability; however, plant functional evidence remains limited. Recent critical approaches emphasize that incomplete genome annotation and short-read ambiguities can inflate intergenic calls. This underscores the need for rigorous orthogonal validation and long-read sequencing [67,70]. Available datasets suggest frequent nuclear enrichment and covariation with nearby gene activity. This finding indicates potential locus-proximal roles that warrant in vivo testing [66]. Community resources such as PlantCircRNA harmonize annotations across many species and experiments. This enables cross-species comparisons and systematic candidate prioritization [69].

## 3. Non-Coding RNAs in Drought

Drought remains a dominant limitation to plant productivity worldwide. Plant ncRNAs constitute a multilayered regulatory system that links the perception of water deficit to the control of gene expression. MiRNAs reshape abscisic acid signaling, reactive oxygen species homeostasis, and root system architecture. Programmable miRNA modules have improved the drought tolerance of major crop species [72,73]. siRNAs, including phased siRNAs, regulate stress-responsive mRNAs and, through RNA-directed DNA methylation, reinforce chromatin states associated with adaptive responses [74]. LncRNAs operate in cis and trans to modulate transcriptional networks under water deficit conditions [75]. CircRNAs are increasingly reported as drought-responsive transcripts across species. Functional studies in maize and *Arabidopsis* support their regulatory potential [64]. NcRNAs coordinate developmental programs with environmental water status and represent tractable targets for breeding climate-resilient plants. A brief summary of the key drought-responsive genes, their regulatory non-coding RNAs (ncRNAs), and the physiological and molecular mechanisms by which they help plants adapt to stress is given in Table 1.

### 3.1. miRNAs in Drought Stress

Drought limits crop productivity worldwide by limiting cell expansion, altering hydraulic conductance and metabolic homeostasis, and activating adaptive programs across tissues. Over the past decade, many studies have shown that miRNAs are central posttranscriptional regulators that fine-tune these programs by repressing mRNA targets involved in hormone signaling, development, and stress defense. Drought-responsive miRNAs display tissue-, stage- and genotype-specific dynamics, with many families conserved across angiosperms but exhibiting species-dependent changes. These small RNAs generally contribute to the trade-off between growth and survival by modulating the auxin and ABA pathways, root architecture, leaf polarity/hydraulics, and oxidative stress responses [72,87].

#### 3.1.1. Auxin and ABA Signaling Modules

**Figure 2:**
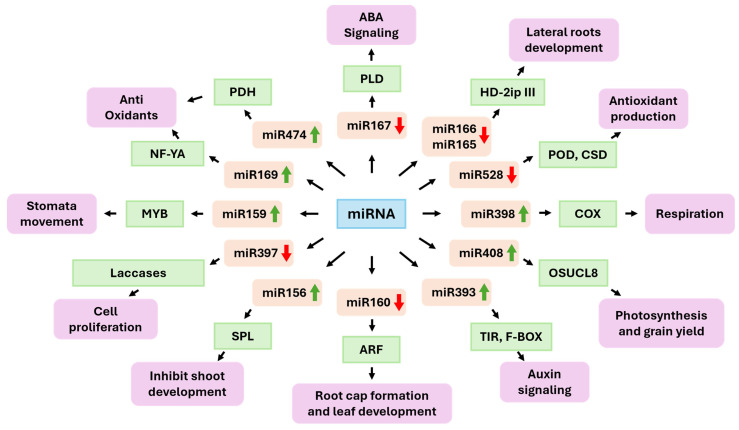
miRNAs involved in the plant response to drought stress. The green arrow indicates up-regulation and the red arrow indicates down-regulation.

The miR393 family has been shown to modulate plant responses to drought, mainly by interacting with auxin F-box receptors, i.e., transport inhibitor response 1 (TIR1) and auxin signaling F-BOX (AFB) proteins, thereby inhibiting auxin perception and signaling (Figure 2). In drought conditions, the overexpression miR393 was observed in various species, including rice, barley, and *Arabidopsis*, leading to downregulation of TIR1/AFB and weaker auxin response pathways. In rice, experiments have shown that expressing miR393 can reduce growth and limit the development of new roots in drought conditions, which is consistent with auxin reduced sensitivity and better use of resources. In barley, the expression profile is linked to variations in stomatal characteristics and greater water use efficiency, suggesting the involvement of this module in development and adaptation to water limitations. However, the direction and magnitude of the response depend on the genotype [88,89]. The miR160–ARF10/16/17 module integrates auxin with other cues. Genetic and physiological analyses revealed that miR160 restrains ARF10/16/17 to modulate seed germination and hypocotyl/root growth. Crosstalk with ABA signaling during stress has been documented [90]. The modulation of miR167, which targets the auxin response transcription factors ARF6 and ARF8, is shaped by the intricate interaction between the auxin and ABA pathways in response to drought [91]. In *Arabidopsis*, miR167 is frequently upregulated under drought conditions, which reduces ARF6/8 levels [92]. This influences root architecture, optimizing water foraging. Conversely, in rice, drought and ABA typically induce the downregulation of miR167. This results in increased ARF6/8 expression and altered adventitious and lateral root formation, highlighting species-specific regulatory patterns. Studies demonstrate that ABA can directly downregulate miR167 in rice, subsequently affecting ARF targets. Thus, the module integrates auxin signaling and ABA responses rather than functioning through completely independent pathways [93,94,95]. Furthermore, a number of studies have reported that the downregulation of miR167, which is associated with drought, is linked to the expression of phospholipase D (PLD). PLD-generated phosphatidic acid promotes ABA signaling and stomatal closure, thereby strengthening drought tolerance. The dual regulation of ARFs and PLDs highlights the adaptability of miR167 in connecting the auxin and ABA pathways [96,97,98,99].

#### 3.1.2. ABA-Linked Transcription Factor Modules

There are also ABA-linked microRNA–transcription factor modules, the most notable of which are the miR159–MYB and miR169–NF-YA modules. ABA coordinates stomatal closure and modulates key transcriptional responses. The miR159-MYB network is key in abscisic acid signaling. In *Arabidopsis*, ABA induces the expression of miR159, which then represses the expression of MYB33/101, resulting in reduced ABA sensitivity in seedlings. Reducing miR159 or stabilizing MYB transcripts increases ABA responsiveness and enhances drought tolerance [87]. In potato, overexpression of miR159-resistant MYB33/65/101 improves drought resilience and increases ABA sensitivity, confirming that this module fine-tunes hormonal signaling [78]. Further downstream, ABI5 integrates the pathway by regulating the expression of genes critical for germination and drought survival [100].

The miR169–NF-YA module plays a critical role in drought stress adaptation and antioxidant defense in plants. Under drought conditions, miR169 is often downregulated, which relieves its posttranscriptional repression of NF-YA transcription factors [101]. Higher levels of NF-YA enhance the expression of genes associated with stress tolerance, including those encoding key antioxidant enzymes such as peroxidases and glutathione transferases, which mitigate ROS accumulation and oxidative damage [77,101]. Studies in *Arabidopsis*, maize and tomato showed that shuttling of the miR169–NF-YA balance supports maintenance of cellular redox homeostasis and improved physiological drought resilience. Moreover, the regulatory network modulates additional biosynthetic and stress-responsive pathways, including coordinating ABA signaling and stress-responsive transcription factors [77,102,103].

#### 3.1.3. Developmental Patterning and Hydraulic Control

Drought stress engages a set of conserved miRNA modules that coordinate developmental patterning and hydraulic control, including the miR165/166–HD-ZIP III pathway, pivotal for specifying plant leaf polarity and vascular differentiation. In rice, sustained depletion of miR166 via Short Tandem Target Mimic (STTM-166) resulted in leaf rolling, altered xylem maturation, and significantly increased drought resistance, demonstrating that miRNA-guided developmental patterning can be used for water loss. Genetic studies in *Arabidopsis* revealed interactions between miR160 and miR165/166, linking auxin and HD-ZIP III circuits in drought-responsive development [104,105]. The conserved miR396–growth-regulating factor (GRF)/growth-inhibiting factor (GIF) module regulates cell proliferation and organ growth. Drought (or PEG/osmotic stress) often elevates miR396 across species, curbing GRF targets and reducing leaf size. Depending on the system, this can reduce the transpiring area, reallocate resources, and modify root growth. Although some reports have attributed reduced stomatal density to miR396 overexpression, a consistent and well-supported outcome controls growth through the repression of GRFs, with context-specific downstream effects on drought performance [106,107]. The miR156–SPL (squamosa promoter binding protein-like) module integrates developmental timing with stress responses. Under drought conditions, increased levels of miR156 lead to a delayed vegetative–reproductive transition, prolonged juvenile traits, and enhanced root development [108]. Maize, rice, and alfalfa overexpression studies have shown improved drought tolerance via higher proline content, more potent antioxidant activity, and altered biomass allocation [109,110]. This regulatory network shows how developmental control can be exploited to increase resilience.

#### 3.1.4. Oxidative Stress and ROS Regulation

A water deficit increases ROS, which are harmful by-products of cellular metabolism. Cu/Zn Superoxide Dismutase 1 and 2 (CSD1/CSD2) are essential Cu/Zn superoxide dismutases that function as key ROS scavengers together with the copper chaperone CCS. They maintain oxidative homeostasis by catalyzing the dismutation of superoxide radicals and thus limiting ROS accumulation [111]. Under drought stress, miRNA398 is commonly downregulated in many dicots and cereals, resulting in increased expression of *CSD1*, *CSD2* and *COX5b*. This bolsters antioxidant defenses and restricts ROS buildup [80]. COX5b notably acts as a mitochondrial electron transport component, modulating mitochondrial respiration in response to water deficit [112]. While the general pattern involves downregulation of miR398 and enhanced antioxidant enzymes during drought or oxidative stress, exceptions have also been reported at the tissue and species level. The expression of miR397 (laccases and cell wall/lignification) and miR408 (plastocyanins and other cuproproteins) also responds to water status. These miRNAs contribute to redox balance and cell wall remodeling [73]. In monocots, monocot-specific miR528 modulates peroxidases, superoxide dismutases, and polyphenol oxidases. In rice, ABA and drought strongly affect miR528 levels, which in turn influence antioxidant production and root development [82]. Broader engineering that co-overexpresses the “copper miRNAs” miR397, miR408, and miR528 enhances stress tolerance in major cereals, highlighting their translational potential.

#### 3.1.5. Osmolyte Accumulation and Metabolic Adjustment

Several datasets report that miR474 is upregulated under drought conditions and is associated with the repression of proline dehydrogenase (PDH) and proline accumulation. As with many miRNA phenotypes, the effects can depend on the stress regime and genotype, and direct functional validation remains comparatively rare [6,113].

#### 3.1.6. Genotype-Conditioned and Translational Dynamics

High-throughput small-RNA profiling across contrasting cultivars and stages revealed that the same miRNA family can be induced in a tolerant genotype but repressed in a sensitive one, with roots and reproductive tissues exhibiting distinct signatures. For example, under terminal drought in rice roots, the families miR166, miR156, miR167, and miR396 were among the most differentially expressed genes between tolerant and sensitive cultivars. Similar genotype-conditioned patterns have been reported in wheat and maize, emphasizing that drought-related miRNA responses are plastic and networked [93,107]. Over the past five years, there has been a shift from descriptive catalogs to functional and translational studies, including target mimicry (STTM), allele-specific editing of miRNA binding sites, and multiplexed modulation of miRNA families. Notable examples include STTM-166 in rice for drought resistance and the overexpression of multiple copper-miRNAs for climate resilience. These studies confirm that miRNA circuits do not act in isolation but integrate hormone signals (ABA/auxin), hydraulic constraints, and redox/metabolic status to orchestrate drought acclimation, balancing growth costs through precise tuning [73,93,104]. Plant miRNAs form a layered network that responds to drought. This network couples hormone signaling (e.g., miR393, miR160, miR167, miR159, and miR169), development and water management (e.g., miR165 and miR166), and ROS/osmolyte homeostasis (e.g., miR398, miR397, miR408, miR528, and miR474). The directionality and effect sizes of these interactions depend heavily on the species, tissue, developmental stage, and type of drought (acute vs. chronic; vegetative vs. reproductive). Therefore, claims about a given family should consider these contingencies and be supported by species- and context-appropriate evidence.

### 3.2. siRNAs in Drought Stress

SiRNAs play pivotal roles in maintaining genome stability, fine-tuning transcriptional activity, and coordinating developmental and stress-responsive pathways. Increasing evidence highlights their critical contribution to plant adaptation under drought stress. They act at multiple levels, from chromatin remodeling to osmolyte metabolism.

**Figure 3:**
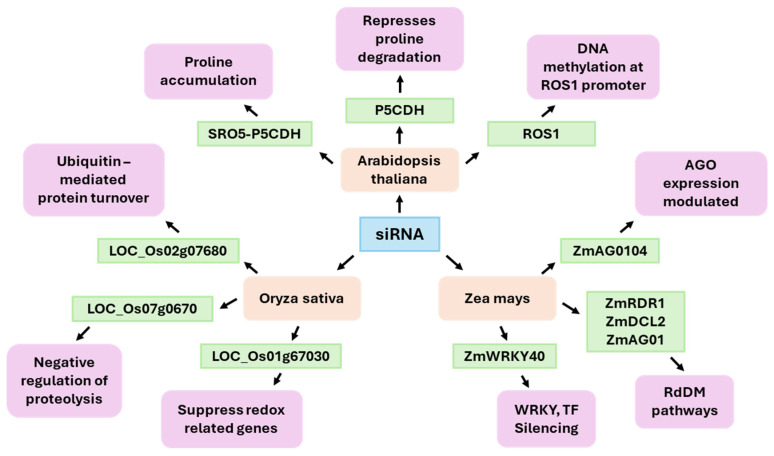
Functions of siRNAs involved in the plant response to drought stress.

*Arabidopsis* has served as a crucial model for elucidating the mechanisms of siRNA-mediated drought responses. The SRO5–P5CDH natural antisense siRNA (nat-siRNA) module is one of the best-characterized pathways (Figure 3). In this system, drought and salt stress induce overlapping transcription of the SRO5 and P5CDH genes, generating double-stranded RNA that is processed into 21-nt nat-siRNAs [30]. These nat-siRNAs specifically target and downregulate P5CDH, which encodes proline dehydrogenase—a key enzyme in proline catabolism. By repressing P5CDH, plants reduce proline breakdown, leading to proline accumulation. Proline accumulation serves both as an osmoprotectant and a reactive oxygen species (ROS) scavenger, directly enhancing tolerance to drought and salinity through improved osmotic and redox balance [30]. Another significant component of drought response relates to the DNA demethylase ROS1 (Repressor of Silencing 1), which counteracts the RdDM pathway. In drought stress, siRNAs directed towards the *ROS1* promoter modulate ROS1 expression, thereby establishing an epigenetic feedback loop that enables plants to adapt demethylation activity in response to variable water availability. This dynamic modulation of DNA methylation establishes epigenetic plasticity that supports long-term adaptation to environmental stress [114,115]. Under drought and salinity conditions, tasiRNA biogenesis components and tasiRNAs (TAS1/2/3, TAS3a, and RDR6 precursors) decrease. The tasiRNA-ARF module then adjusts the dosage of ARF3 and ARF4 to sustain floral organ patterning and reproductive success under stress. These findings demonstrate how 21-nt siRNA pathways interact with hormone response networks to balance growth and stress resilience [116]. In maize, high-coverage small RNA (sRNA) profiling under water deficit conditions shows a pronounced skew toward siRNA upregulation [117]. In maize, siRNA responses to drought encompass the canonical RNA-directed DNA methylation pathway and additional regulatory interactions with stress-responsive transcription factors. Under drought stress, specific siRNAs were implicated in the posttranscriptional regulation of ZmWRKY40, a transcription factor involved in ABA signaling and defense responses [118]. Moreover, specific siRNAs targets genes encoding core silencing machinery components—such as ZmAGO1, ZmAGO104, ZmDCL2, and ZmRDR1—implying the presence of autoregulatory feedback loops that sustain RNA silencing capacity during environmental stress [119]. This dynamic siRNA-mediated regulatory landscape underscores the intricate epigenetic and posttranscriptional mechanisms underlying drought resilience in maize. In rice, drought stress induces the production of siRNAs that overlap with numerous protein-coding genes linked to proteolysis and the regulation of oxidative stress. The identification of specific loci siRNA in the proximity of genes such as *LOC_Os02g07680*, *LOC_Os07g0670*, and *LOC_Os01g67030* was undertaken, with the expression of these loci exhibiting a negative correlation with transcripts encoding proteins involved in ubiquitin-mediated protein turnover and redox-related processes. Despite the paucity of direct evidence for siRNA-guided mRNA cleavage, functional associations suggest that these siRNAs, induced by drought, help suppress the expression of key genes involved in proteolytic processes and oxidative stress, and contribute to the maintenance of proteostasis and cellular redox balance during periods of water deficit [120]. This coordinated posttranscriptional regulation likely reduces protein degradation and ROS accumulation, supporting physiological adaptation to drought. Cereal models reveal lineage-specific patterns. In barley, small RNAome surveys have shown that repeat-associated small interfering RNAs (rasiRNAs) tend to be downregulated by drought. This contrasts with maize, which tends to upregulate siRNAs. These differences likely reflect species- and genome-architecture-specific transposable element (TE) landscapes and RdDM organization [121]. In sugarcane, short-term water depletion increases the abundance of 22-nt sRNAs and induces the production of siRNA candidates consistent with the genotype across tolerant and sensitive backgrounds. Size redistribution of TE-derived sRNAs (including LTR-gypsy-related signals) has been observed in tolerant materials, which is consistent with stress-induced remodeling of sRNA production from repetitive regions [122]. At the chromatin interface, hc-siRNAs often mark mCHH islands at gene–TE boundaries and help establish RdDM boundaries. Studies in maize and *Arabidopsis* have demonstrated that siRNA-directed methylation maintains euchromatin/heterochromatin transitions near genes. Drought disturbs these systems, causing increases or decreases in methylation depending on the genotype, tissue, and stress regime. Current reviews focusing on crops support the view that drought stress uses RdDM to modulate gene expression and splicing while establishing forms of stress memory in some contexts [123]. One practical implication is that siRNAs serve as markers and mediators of drought responses. In maize and sorghum, siRNA variation coincides with shifts in the expression of genes involved in hormone signaling, development, and osmotic adjustment pathways [124,125]. In sugarcane, siRNA candidates consistently distinguish between tolerant and sensitive cultivars under water deficit conditions [122]. Thus, integrating the siRNAome, methylome, and transcriptome profiling offers a way to identify regulatory nodes for breeding and engineering drought resilience, considering that responses are highly context-dependent and temporally dynamic.

### 3.3. CircRNAs in Drought Stress

**Figure 4:**
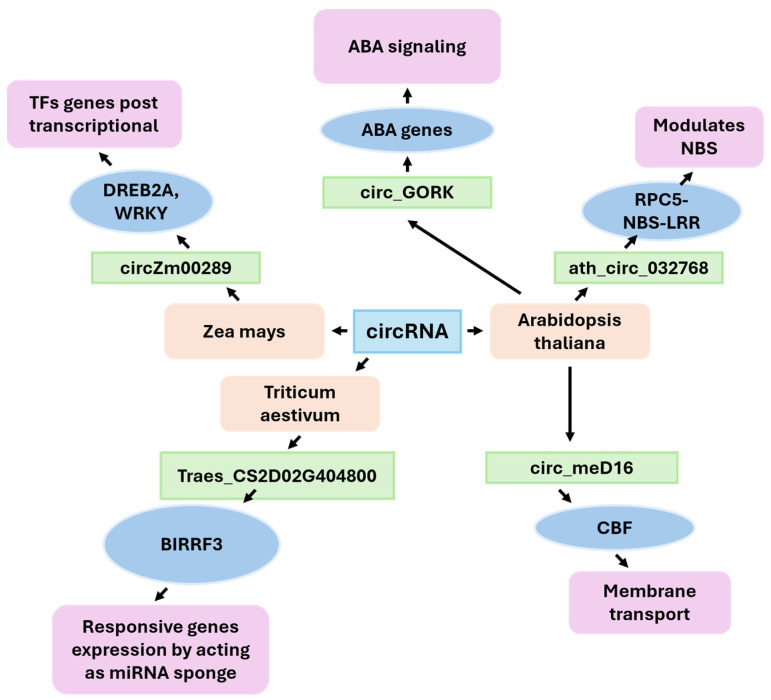
The importance of circRNAs in plants after drought.

CircRNAs are a class of covalently closed RNA molecules that are generated through a process known as back-splicing, which involves the formation of a bond between the 5′ and 3′ ends of a pre-mRNA molecule. The circular configuration of these transcripts offers enhanced stability, a property that safeguards them from the process of exonucleolytic degradation. In plants, recent studies have identified circRNAs as key posttranscriptional regulators in developmental pathways and abiotic stress responses, including drought adaptation. CircRNAs have been confirmed to play a role as competitive endogenous RNAs (ceRNAs). By acting as molecular sponges that sequester specific miRNAs, circRNAs modulate the abundance and activity of stress-responsive target mRNAs [64,65,126,127]. At the systems level, the repertoire of circRNAs expands under drought conditions in both monocots and dicots. For example, thousands of high-confidence circRNAs have been cataloged in maize and *Arabidopsis*, many of which exhibit drought-responsive dynamics and host gene enrichment in pathways such as calcium signaling, hormone responses, and redox regulation [126]. Notably, the functions of several plant circRNAs have been tested, linking specific molecules to altered drought performance. In *Arabidopsis*, the circular RNA ath-circ032768 forms a validated ceRNA module with miRNA472 and RPS5, a CC-NBS-LRR protein involved in stress adaptation. Overexpressing circ032768 (or silencing miR472) increases drought tolerance, which is consistent with the sequestration of miRNAs that derepress *RPS5* and increase the expression of genes associated with drought, such as *DREB2A*, *RD29A*, and *RD29B* [65]. This study provides direct genetic evidence that a plant circRNA can regulate a small RNA–immune receptor axis to promote dehydration tolerance. Another example from *Arabidopsis*, circGORK, which is derived from the guard-cell outward-rectifying K^+^ channel locus, confers hypersensitivity to ABA and supports drought tolerance in transgenic lines. These findings suggest that circRNAs function in stomatal control and guard-cell signaling [126]. These cases substantiate a recurrent theme: drought-responsive circRNAs modulate small RNA availability and/or stress response nodes, resulting in altered water use and protection against cellular damage. In maize, drought-induced circRNAs are distributed throughout the genome. A comprehensive study identified 2174 circRNAs in seedlings and determined that many are differentially expressed during water deficiency [64]. CircRNAs hosted by calcium-dependent protein kinase (CPK) and cytokinin oxidase/dehydrogenase (CKX) genes are significantly associated with seedling drought tolerance. These findings suggest a functional link between circRNA biogenesis and key hormonal and calcium signaling pathways [3]. A recent analysis of roots revealed that drought-enriched circRNAs coincided with transposon content (LINE retrotransposons) and local chromatin features. This analysis also highlighted technical criteria to distinguish robust circRNAs from artifacts [64]. Another candidate, circZm00289, was associated with DREB2A and WRKY transcription factors, key regulators of dehydration-responsive gene expression (Figure 4) [69]. In wheat, circRNA responses to drought exhibit genotype specificity. In total 1409 circRNAs were cataloged from wheat seedlings exposed to water deficit—820 circRNAs in the drought-resistant variety and 722 in the drought-sensitive one. Expression analysis revealed a striking pattern: over 88% of circRNAs were upregulated in the resistant variety under drought, whereas more than 60% of circRNAs were downregulated in the sensitive genotype [86]. Crucially, several regulatory circRNA–miRNA–mRNA modules were characterized. The tae-miR1122b-3p module involves aquaporin (TraesCS2D02G404800), which plays a fundamental role in water transport, and OsBIERF3 homologs, which enhance both salt and drought resilience. Another, the tae-miR9664-3p module, centers on peroxidase and ethylene-responsive transcription factor genes, implicating circRNAs in reactive oxygen species homeostasis and ethylene signaling—two pathways vital for stress adaptation [86].

The PlantCircRNA database compiles plant circRNAs from various species and allows users to query back-splice junctions, expression levels, and predicted interactions [69]. Recent reviews summarize the best practices for plant circRNA discovery, including artifact filtering and validation methods such as RNase R resistance, junction PCR/Sanger confirmation, and functional tests [66,69,127,128]. These resources are essential for standardizing drought-induced circRNA catalogs and prioritizing candidates for functional assays. Collectively, the convergent observations that drought induces changes in circRNA expression and that specific circRNAs modulate ABA sensitivity, stomatal behavior, immune/stress transcription factor (TF) networks, and ROS management and that circRNA signatures are genotype dependent suggest that circRNAs constitute a regulatory layer in plant drought adaptation. Function-validated cases (ATH-CIRC032768, CIRCGORK, and CIRCMED16) provide blueprints for the use of circRNAs or their modules (circRNA-miRNA–mRNA) in breeding or engineering. However, transitioning from association to application will necessitate broader functional validation across crops, careful analysis of off-target small RNA effects, and field-relevant water use and yield outcome testing.

### 3.4. LncRNAs in Drought Stress

**Figure 5:**
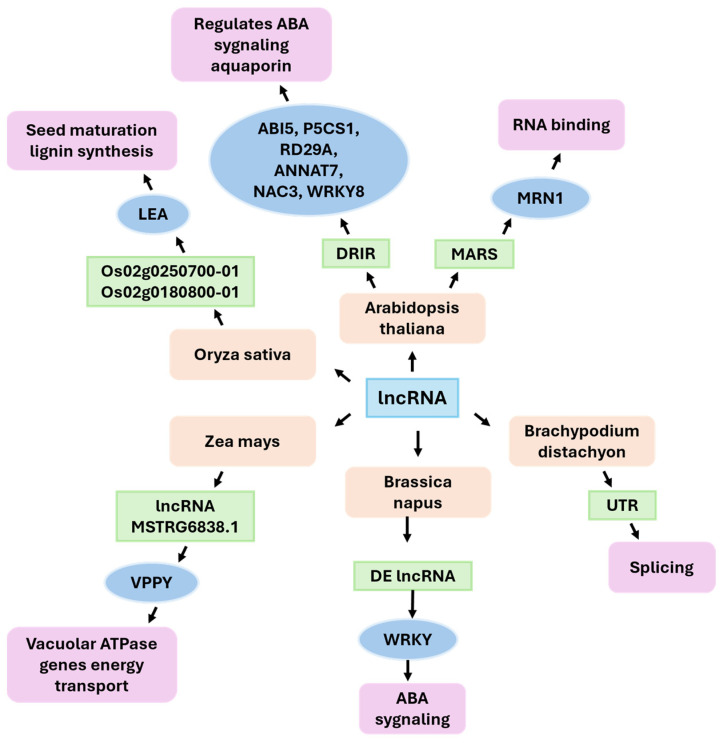
Functions of lncRNAs identified in various plants.

LncRNAs are pervasive regulators of plant responses to water deficit. They act through cis and trans control, lncNATs, RNA–protein interactions, and ceRNA circuits. In crops, drought reshapes the expression programs of lncRNAs in a tissue-, stage-, and genotype-dependent manner. Several lncRNAs link regulatory layers, such as hormone signaling, the chromatin state, and membrane transport, with physiological drought tolerance [129,130,131]. Reanalysis of public RNA sequencing (RNA-seq) datasets from rice revealed thousands of stress-responsive lncRNAs in shoots and roots [132]. Six drought-induced lncRNAs (DRIL1–DRIL6) were prioritized and experimentally validated. DRIL1–DRIL5 are intergenic, whereas DRIL6 is a NAT (Figure 5). The transient overexpression of selected DRILs upregulates canonical drought/ABA marker genes, supporting a positive role in drought signaling [129]. Furthermore, independent strand-specific surveys revealed that lncNATs are correlated with drought responses in both cultivated *Oryza sativa* and wild *Oryza nivara*, underscoring the contribution of antisense regulation to adaptive variation in rice [132]. Additional studies in rice have revealed that natural antisense transcripts (NATs) are frequently located near late-embryogenesis-abundant (LEA) genes and genes related to metabolic pathways responsive to drought. These findings are consistent with regulatory modules that govern seed maturation, lignin biosynthesis, and ABA signaling—key processes in drought adaptation [133]. Multistage, multitissue profiling in maize identified thousands of drought-responsive lncRNAs with distinct developmental trajectories. For example, MSTRG.2834.1 and MSTRG.43642.1 are significantly differentially expressed in reproductive tissues during drought. An intergenic lncRNA, MSTRG.6838.1, is cis-correlated with the adjacent *VPP4* (*GRMZM2G028432*) gene, which encodes a vacuolar H^+^-pumping ATPase subunit. Both transcripts are repressed by drought across tissues, identifying this lncRNA-pump pair as a candidate module that links vacuolar homeostasis with drought responses [40,130]. Systems studies focused on roots further integrate lncRNAs with histone modifications, the recombination landscape, and survival-associated co-expression modules, thereby reinforcing the centrality of these networks in maize drought tolerance [134]. In cassava, strand-specific RNA-seq revealed 833 high-confidence lncRNAs, 124 of which respond to polyethylene glycol (PEG)-simulated water deficit [135]. Functional inference connected drought-responsive lncRNAs with hormone metabolism and stress pathways. A notable cis-acting example is TCONS_00060863, which is located near a CYP707A-type ABA 8-hydroxylase. Coordinated expression suggests a connection to ABA catabolism during dehydration. Additionally, several cassava lncRNAs are predicted to act as microRNA (miRNA) decoys, suggesting the existence of ceRNA layers that may modulate modules such as miR156/miR172 during stress [135]. Surveys of the rapeseed genome under drought stress have identified hundreds of differentially expressed lncRNAs, many linked to hormone signaling, redox balance and WRKY transcription factors. Co-expression network analyses suggest that specific lncRNAs regulate distinct metabolic and hormone pathways, with functional enrichment revealing involvement in signal transduction and drought adaptation in the crop. Some lncRNAs co-express or co-locate with genes involved in ABA and auxin signal transduction, indicating a regulatory interface between hormone responses and stress signaling [6,136,137]. A study of the *Brachypodium distachyon* genome revealed hundreds of drought-responsive long non-coding RNAs linked to diverse biological processes [138,139]. A large number of *Brachypodium* ncRNAs also regulate genes at the posttranscriptional level, often affecting the splicing of protein-coding genes (“UTR/splicing” pathways). Introns, alternative acceptor/donor sites and complex splicing events involving long non-coding RNAs contribute to changes in gene expression under water-deficit conditions. The landscape of lincRNA diversity and regulatory influence mirrors drought-responsive modules in related cereal crops, confirming the importance of lincRNA-mediated control of development and stress responses in grasses [139,140]. In addition to these examples, cross-species syntheses confirm that the numbers reported a decade ago (e.g., ~664 drought-responsive lncRNAs in maize and ~98 in rice) are reproducible anchors upon which newer datasets were built while also expanding the catalog and adding regulatory annotations (cis, ceRNA, and precursor) [11,131,141,142]. Recent crop-centric meta-analyses suggest that lncRNAs often converge on ABA signaling, auxin crosstalk, and ROS homeostasis. Many of these lncRNAs are located within QTLs or harbor SNPs associated with drought survival [11,134,142].

Although direct causal roles are relatively rare, functional gains are emerging. For example, the *Arabidopsis* lincRNA DRIR enhances drought and salt tolerance, modulates ABA/water transport gene expression and provides a conceptual blueprint for engineering [143]. In parallel, stress-responsive lncRNA networks often intersect with membrane energization—for example, the maize MSTRG.6838.1–vpp4 locus identifies vacuolar pumps as lncRNA-linked effectors. Transgenic studies in other systems have demonstrated that increasing vacuolar proton pumps (e.g., AVP1/V-PPase in cotton and VHA-A in apple) improves water-use efficiency or drought performance when tested in *Arabidopsis*. These findings suggest that these proteins are plausible mechanistic targets for lncRNA-mediated control [144,145]. In *Arabidopsis*, lncRNA MARS has been observed to participate in the regulation of mineral cluster gene expression in response to ABA signaling, which affects root sensitivity to osmotic stress [145]. Plant lncRNAs constitute a stress-responsive, evolvable layer that coordinates hormone signaling, chromatin, and membrane physiology. This finding offers actionable entry points for improving drought resilience in cereals and root crops.

## 4. Bioinformatic Identification of ncRNAs

### 4.1. miRNAs

**Figure 6:**
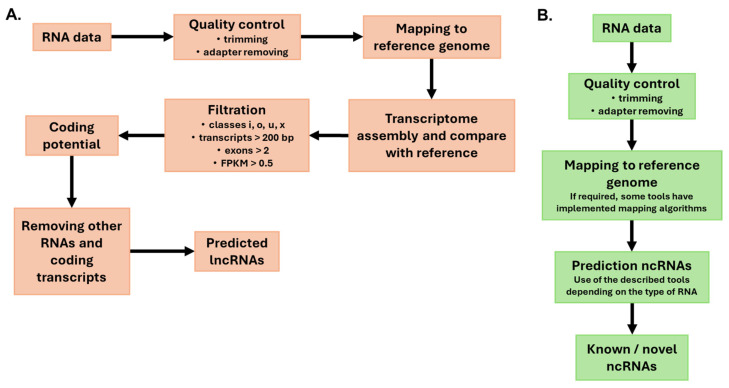
Bioinformatics pipeline analysis of (**A**) Long non-coding RNAs; (**B**) small RNAs and circRNAs.

Robust identification of plant miRNAs via small RNA sequencing (sRNA-seq) requires rigorous preprocessing, genome-aware alignment, and adherence to community standards to reduce false positives (Figure 6). After adapter removal, length and quality filtering, and collapse of identical reads, high-stringency workflows map sRNAs to the reference genome and evaluate candidate hairpin precursors. These workflows also require evidence consistent with Dicer-like processing, including a dominant 20–22 nt species accompanied by the corresponding miRNA* and appropriate duplex features [146,147,148]. Contemporary tools explicitly implement the 2018 plant miRNA criteria to mitigate plant misannotation and encourage validation with degradome (PARE) evidence when available.

Among the plant-specific predictors, miRDeep-P2, an upgraded version of miRDeep-P, is widely used [146]. It overhauls the filtering and scoring processes and integrates updated plant criteria. miRDeep-P2 supports Bowtie/Bowtie2 for mapping and achieves superior speed on large genomes without sacrificing accuracy. A companion methods article details its end-to-end operation on *Arabidopsis*, rice, tomato, maize, and wheat libraries, emphasizing its strict evaluation of precursors and its replication across libraries [146]. Although older, miRPlant remains a practical choice for users who prioritize an interactive interface. It provides an alternative, self-contained, GUI-based workflow derived from the miRDeep family that identifies known and novel plant miRNAs from sRNA-seq [149].

In terms of current best practices, miRador enforces the community’s revised plant criteria, predicts inverted repeats at the genome scale, maps sRNA reads to identify candidate precursors, and integrates target prediction and PARE data to estimate precision [147]. Direct comparisons revealed that miRanda matched or exceeded the precision of miRDeep-P2 and ShortStack while running substantially faster across the *Arabidopsis*, rice, maize, and wheat libraries. ShortStack remains valuable for locus-centric annotation in complex plant genomes [150]. Its local-weighting algorithm places multimapping small RNA (sRNA) reads more accurately than random or exclusion strategies do. This is crucial for repetitive plant DNA and distinguishing miRNAs from abundant siRNAs [148].

Deep learning approaches are beginning to influence the discovery of plant miRNAs [151]. SRICAT implements convolutional neural networks that use raw sequences and precursor structural features to identify known and novel plant miRNAs [151]. It is packaged with a graphical interface and benchmarking, demonstrating competitive or improved accuracy compared with conventional pipelines. These models are applicable when precursor heterogeneity or low expression complicates rule-based scoring. However, they should be used alongside curated references and community criteria to maintain interpretability.

The choice of the annotation backbone and reference database is equally important. For plants, PmiREN2.0 provides a curated, function-oriented knowledge base that aggregates high-quality miRNA annotations, targets, and metadata across more than 170 species and is routinely updated [152]. sRNAanno complements this resource by offering reannotated miRNAs across 143 plant genomes. It also provides extensive phasiRNA and hc-siRNA loci annotated under stringent settings. Cross-checks against PmiREN and community pipelines indicate that sRNAanno is more complete and reliable than legacy submissions are [153]. However, relying solely on miRBase can be problematic for plants because submission-driven variability has historically permitted misannotations [154]. Therefore, current workflows often prioritize PmiREN/sRNAanno for discovery and benchmarking while reporting to miRBase for community interoperability.

Several technical considerations consistently improve plant miRNA calls across tools. First, they map to the genome rather than transcriptomes or hairpins to discover bona fide precursors and assess the local small RNA context. Most pipelines support Bowtie/Bowtie2 with parameters tailored to short, ungapped reads [146,147]. Second, replication and abundance thresholds are enforced across biological libraries, and detectable miRNA* reads are needed to discriminate miRNAs from siRNA-rich loci [146,147,153]. The third step involves inspecting multiple mappings. Algorithms that leverage the local genomic context (e.g., ShortStack) reduce placement bias, especially in repetitive regions [148]. Fourth, the predicted targets were validated via the degradome/PARE when possible, and those signals were incorporated post hoc or within the pipeline (as in miRador) to estimate precision [147]. Finally, integrated suites such as sRNAbench (sRNAtoolbox 2022 update) provide modern annotation backbones (including PmiREN for plants), isomiR handling, and containerized deployments that facilitate reproducible analyses across large cohorts [155].

Contemporary plant miRNA identification emphasizes stringent precursor evidence, careful handling of multiple mapping reads, and validation against curated plant-specific resources. Adopting pipelines that implement the revised criteria and, when possible, integrate PARE signals while benchmarking against PmiREN2.0 and sRNAanno minimizes false discovery and yields annotations suitable for comparative and functional genomics in crop and model species.

### 4.2. siRNAs

The computational discovery of plant siRNAs relies on sRNA-seq coupled with locus-level annotation and the stringent handling of multiple mapping reads (Figure 6). Popular frameworks include the UEA sRNA Workbench, which uses the SiLoCo module to group sRNAs into genomic loci and compare their abundance across conditions. This finding supports exploratory and differential analyses of siRNA populations [156,157]. ShortStack provides an integrated pipeline that defines sRNA loci de novo, quantifies them, and addresses the pervasive multimapping issue through a local-weighting placement strategy. This dramatically improves the precision of siRNAs originating from repetitive regions and transposable elements [148,150].

Study-specific rules are often applied to identify siRNAs from sRNA-seq datasets. For example, Ge et al. reported that candidate pairs in maize embryogenic calli form a canonical Dicer-like duplex with two-nucleotide 3′ overhangs and that at least one strand accumulates at least five reads [158]. Then, they integrated differentially expressed siRNAs and their targets with DNA methylation and expression profiles [158]. Subsequent work in Chinese cabbage under heat stress adopted the Ge et al. criteria, identifying hundreds of differentially expressed siRNAs and thousands of predicted targets, with ~795 targets shared across time points [159]. These examples illustrate standard practices, such as read–count thresholds, duplex features, and multiomic corroboration.

Newer tools have expanded beyond locus calling. SmallDisco identifies siRNAs as short reads that map antisense to annotated genomic features, such as exons and mRNAs. Notably, 3′ nontemplated tailings were quantified via Tailor, which helps assess siRNA maturation and stability [160]. At the resource level, sRNAanno provides uniformly curated annotations of plant small RNAs, including hc-siRNA and phasiRNA loci. This enables better benchmarking and cross-species comparison [153]. Similarly, a large-scale, uniform reannotation of 47 plant genomes standardized sRNA locus definitions revealed the prevalence of 24-nt hc-siRNA and 21-nt phasiRNA loci among siRNA producers. This reannotation offers recommended parameters for future studies [161].

In practice, robust siRNA identification should trim/adaptor-filter reads, remove r/t/sn/snoRNAs, and map with sensitive aligners. It should also define loci genome wide (e.g., ShortStack or SiLoCo), explicitly treat multiple mappers (ShortStack’s placement), call siRNA candidates on the basis of duplex/size criteria and minimal abundance, integrate evidence (e.g., RdDM marks and phasing, when relevant), and validate them via differential analysis and target prediction. Ideally, this process is supported by mRNA or methylome data.

### 4.3. lncRNAs

State-of-the-art plant lncRNA discovery typically follows a reference-guided “new Tuxedo” logic involving short-read alignment, transcript assembly, reference comparison, and multilayer filtering. Modern implementations use HISAT2 version 2.2.1 for spliced alignment and StringTie2 version 3.0.1 for sensitive assembly, including long-read support [162,163,164]. After the assemblies from each sample are merged, gffcompare contrasts the merged transcriptome with the reference transcriptome to assign class codes. Candidates are usually drawn from U (intergenic), X (antisense exonic overlap), I (fully intronic), and O (other same-strand overlap). Some studies also keep J for multiexon novel junction matches. Size filters retain transcripts ≥200 nt, and most plant workflows require ≥2 exons to limit artifacts. Some workflows optionally allow monoexon lncRNAs when supported by stricter abundance/conservation evidence [165,166].

Expression thresholds are applied to reduce the stochastic background. Recent plant pipelines commonly use an expression threshold of FPKM/TPM ≥ 0.5 (or occasionally ≥0.1 for multiexon models) before coding-potential screening [167,168]. The coding potential is evaluated via ensemble strategies alongside legacy classifiers (CPAT, CPC2, CNCI, and PLEK). Newer frameworks either retrain models on curated plant sets or adopt deep learning. The Plant-LncPipe retrained the CPAT and PLEK via plant data [164]. It was then benchmarked against CPC2, CNCI, LncADeep, and RNAplonc. The results revealed substantial gains in plant-specific accuracy. The pipeline also integrates transcript length, open reading frame (ORF) limits, and similarity filtering against UniProt, Pfam, and Rfam. This process purges residual coding and structured RNAs [164]. Complementary deep learning tools, such as DeepPlnc (a bimodal convolutional neural network [CNN] that uses sequences and structures) and LncDC (an XGBoost model that uses sequence, structure, and protein translation features), provide high accuracy for plant lncRNA classification. These tools can be secondary consensus layers [169,170].

High-confidence sets remove housekeeping and structural RNAs (rRNA, tRNA, snRNA, and snoRNA), filter transposable-element-derived transcripts, and scrutinize multiple mapping reads. Practical and reproducible workflows have emerged, such as ICAnnoLncRNA (Snakemake) for plants, which package mapping, assembly, filtering, evaluation of coding potential (e.g., LncFinder), transposable element (TE) masking, genomic classification, and database cross-checks [4]. Recent case studies explicitly document the selection of U/O/I/X (±J) classes, transcripts ≥ 200 nt long with ≥2 exons, expression cutoffs, and ORF constraints (e.g., ORF < 300 nt). These studies also document the exclusion of transcripts via Pfam, Rfam, and NR, yielding robust catalogs amenable to differential expression and network analyses [166,167,168].

In summary, modern identification of plant lncRNAs emphasizes StringTie2-quality assemblies rather than reference annotations by gffcompare, stringent length/exon/abundance filters, plant-aware coding-potential ensembles (preferably retrained), and deep-learning classifiers. It also emphasizes the comprehensive removal of coding, housekeeping, and transposable element (TE) confounders. Using recent pipelines, such as Plant-LncPipe or ICAnno LncRNA, helps standardize these steps and improves cross-study comparability.

### 4.4. CircRNAs

The computational identification of plant circRNAs focuses on detecting back-spliced junctions (BSJs) in RNA-seq reads, which are then quantified and functionally annotated (Figure 6). Since plant genomes differ from animal genomes in terms of splicing signals, gene copy number, and circRNA length distributions, plant-aware pipelines are recommended [171]. CircPlant is a dedicated framework that maps reads via BWA-MEM and integrates a plant-modified CIRI2 to identify BSJs. CircPlant then predicts circRNA–miRNA interactions via TargetFinder and TAPIR, constructs circRNA–miRNA–mRNA (ceRNA) networks, and performs GO enrichment to infer functional themes. Comparative tests on simulated and real plant datasets indicate that CircPlant has higher precision and F1 scores than competing tools do [171].

Among the plant-specific detectors, PcircRNA_finder focuses on exonic circRNAs and implements a three-module design: Catcher (BSJ discovery via chiastic clipping and fusion detection), Annotator (context from gene models), and Filter (quality controls to reduce false positives common in high-copy-number plant loci) [172]. PCirc is a complementary machine-learning route that trains a random forest classifier on rice circRNA versus long non-coding RNA features, such as k-mers, open reading frames (ORFs), and junction coding. PCirc achieved >0.99 accuracy in internal tests and >0.8 accuracy in tests across *Arabidopsis* and maize. This makes PCirc a practical postcalling validator or primary predictor when appropriate training data are available [173].

General-purpose detectors are still commonly used in plant studies. CIRI2, which uses multiseed matching with an adapted maximum-likelihood scorer, is effective for BSJ detection and is used in several plant pipelines [174]. CIRCexplorer2 uses chimeric alignments (e.g., from STAR) and provides extensive annotation tools [175]. DCC provides STAR-based calling with replicate-aware filtering and a paired CircTest module for differential abundance analysis [176]. Although the legacy find_circ script is fast and straightforward, it benefits from downstream corroboration. It identifies circRNAs by extracting 20-nt anchors from unmapped reads, realigning them, and retaining BSJ-supporting splits [177,178].

CIRIquant was used for quantification and differential expression. It explicitly models treatment biases (e.g., uneven RNase R enrichment) and corrects BSJ undercounting. This enables more reliable circRNA expression estimates [179]. Owing to tool-specific biases, ensemble strategies, such as CirComPara2 (automated multicaller integration) and SRCP (annotation-then-quantification), often improve recall without sacrificing precision. These strategies are advisable when the sample size permits [180,181]. Best-practice guidelines recommend poly(A)- or rRNA-depleted libraries for discovery, explicit reporting of mapping and BSJ filters, and orthogonal validation via divergent-primer RT–PCR with or without RNase R. Caution should be exercised when interpreting ceRNA inferences [182].

The Find_circ tool [146] is widely used for circRNA prediction. This script, however, requires the raw RNA-seq data to be preprocessed independently via additional software. The filtered data need to be mapped to a reference genome, and then 20-nt fragments must be selected from the unaligned reads and mapped back to the reference. The data prepared in this way were used in find_circ to predict potential circRNAs [183,184,185,186].

The robust discovery of plant circRNAs typically combines a plant-aware caller (e.g., CircPlant or CIRI2-based workflows) with quantification-focused tools (e.g., CIRIquant) and, when feasible, ensemble callers to cross-validate backsplicing junctions (BSJs). Functional follow-up, including miRNA–target prediction and ceRNA networks, should be accompanied by transparent parameterization and validation to ensure reliability across diverse plant genomes.

## 5. Perspectives for the Breeding of Climate-Resilient Cultivars

Non-coding RNAs provide actionable pathways for climate-resilient breeding when combined with contemporary genomics and precision editing. At the small RNA level, miRNA circuits can be scaled up: the coordinated overexpression of “copper miRNAs” (miR397, miR408, and miR528) in maize and wheat increases drought and cold tolerance, demonstrating that the simultaneous modulation of conserved nodes can increase the resilience of major cereals [73]. Similarly, allele-precise editing of miRNA pathways is feasible. CRISPR/Cas12a has been used to mutate *MIR* genes, and Cas9 disruption of miRNA target sites derepressed selected transcripts without rewiring the entire network—an appealing strategy for uncoupling stress tolerance from yield penalties by fine-tuning specific interactions [187,188]. The siRNA-RdDM interface places ncRNA control at the chromatin level. Drought alters DNA methylation in crops, and the plant siRNA landscape highlights the prevalence of 24-nt populations associated with RdDM [22,123]. These findings support the development of targeted epigenome editing and markers from stress-responsive methylation contexts as complementary strategies for achieving long-term adaptation [9,189]. In addition to sRNAs, long non-coding RNAs and circular RNAs are emerging as targets. However, their translation benefits from rigorous validation, such as long-read support and degradome/AGO-CLIP, as well as functional assays that confirm causality across environments [9,64]. Deployment in elite germplasms is becoming more practical. Haploid-inducer-mediated genome editing (HI-Edit/IMGE) accelerates the introgression of edits into breeding lines. Moreover, virus-induced genome editing (VIGE) is a tissue culture-sparing delivery method for rapid trait prototyping [190,191]. Strategically integrating ncRNA-informed edits and markers with genomic selection, multiomics phenotyping, and multienvironment field trials is essential for balancing resilience, productivity, and quality.

NcRNA-guided engineering, which includes multiplex miRNAs, MIR/target-site editing, RdDM-aware epigenome interventions, and validated lncRNA/circRNA nodes, can define a roadmap for cultivating climate-resilient cultivars.

## 6. Conclusions

Non-coding RNAs are critical regulators of plant responses to drought stress, contributing significantly to plant survival and resilience under adverse environmental conditions. Recent evidence highlights their essential role in regulating gene expression in physiological and biochemical adaptations to water deficit. This regulation is important for cereal crops, which are essential for global food security and are especially vulnerable to the increasing incidence and severity of droughts driven by rapid climate change. The dual challenges of a growing worldwide population and dynamic climatic conditions require the development of drought-resistant crop varieties. In this context, non-coding RNAs, including small RNAs, long non-coding RNAs, and circular RNAs, play indispensable roles in orchestrating adaptive plant responses. These RNAs control gene networks involved in water uptake, transport, conservation, and stress signaling pathways, facilitating the fine-tuned regulation of drought tolerance. Owing to high-throughput RNA sequencing technologies and thorough functional analyses, numerous ncRNAs have been identified in various plant species. However, the functional characterization of many genes remains incomplete, representing a significant knowledge gap.

Small non-coding RNAs, particularly microRNAs, play a role in localized gene regulation and systemic signaling by translocating between plant tissues, such as roots and shoots. This enhances coordinated stress responses. For example, miR399 modulates phosphate homeostasis through systemic movement within plants. Additionally, specific miRNAs are induced by drought and salinity stresses, which modulate the expression of target genes and transcription factors integral to stress resistance mechanisms. Long non-coding RNAs contribute additional layers of regulation by acting as molecular decoys or mimics for microRNAs, establishing complex regulatory networks that fine-tune gene expression. Despite the accumulation of data on ncRNA functions, significant questions remain regarding their intracellular transport mechanisms, stability during long-distance movement, and precise molecular interactions. Methodical and integrated studies are essential to fully elucidate these aspects, thereby enabling the effective translation of ncRNA knowledge into crop improvement strategies to increase drought resilience.

In brief, non-coding RNAs represent encouraging pathways for enhancing our comprehension of plant drought adaptation. Their regulatory potential could be exploited in modern breeding programs and biotechnological approaches to develop crop varieties with enhanced drought tolerance, promoting agricultural sustainability in the face of environmental change.

## Figures and Tables

**Table 1:** Summary of the key drought-responsive genes, their regulatory ncRNAs and the physiological and molecular mechanisms help plants adapt to stress.

Gene/Module	ncRNA Regulator	Core Function	Physiological/Molecular Mechanism	Ref
RD29A, RD22, DREB2A	miRNA, tasiRNA	Stress signaling; TF activation	ABA-dependent/independent pathways, transcriptional control of drought-responsive genes, adaptation to water deficit	[76]
P5CDH (Proline DH)	nat-siRNA (SRO5–P5CDH)	Proline catabolism	Suppression of proline degradation, osmoprotection, ROS scavenging	[30]
NF-YA	miR169	Transcription factor; antioxidant	Enhances antioxidant enzyme expression, improves drought tolerance	[77]
MYB33/101/65	miR159	Transcription factor; ABA signal	Fine-tunes ABA sensitivity, coordinates seedling growth under drought	[78]
ARF6/8/10/16/17	miR167, miR160	Auxin signaling	Regulates root architecture, auxin–ABA crosstalk, optimizes root adaptation	[79]
CSD1, CSD2, COX5b	miR398	ROS detox enzymes; ETC component	Maintains ROS balance, enhances mitochondrial respiration and antioxidant capacity	[80]
Laccase, Peroxidase, PPO	miR397, miR398, miR528	Cell wall & redox	Lignin biosynthesis, cell wall reinforcement, antioxidant defense	[81,82,83]
SPL, WRKY, NAC	miR156, tasiRNA, circRNA	TFs, stress signaling	Delays vegetative-to-reproductive transition, regulates developmental and stress pathways, enhances drought resilience	[84,85]
Aquaporin, OsBIERF3	circRNA/miRNA module	Water/ion transport, TFs	Regulates water transport (aquaporins), ethylene/stress factors, sustains water-use efficiency	[86]
RPS5 (CC-NBS-LRR)	ath-circ032768/miR472	Resistance protein; ceRNA	CircRNA–miRNA–mRNA module derepresses stress-adaptive genes, enhances drought tolerance	[65]

## Data Availability

No new data were created or analyzed in this study. Data sharing is not applicable to this article.
